# Effect of mechanical stresses on viral capsid disruption during droplet formation and drying

**DOI:** 10.1016/j.colsurfb.2023.113661

**Published:** 2023-11-22

**Authors:** Holly Coleman, J. Saylor Perez, Daniel K. Schwartz, Joel Kaar, Robert L. Garcea, Theodore W. Randolph

**Affiliations:** 1. Department of Chemical and Biological Engineering, University of Colorado Boulder, CO 80303; 2. Department of Molecular, Cellular, and Developmental Biology, University of Colorado Boulder, CO 80303

**Keywords:** spray drying, virus, airborne, transmission, droplet, DNA

## Abstract

Identification of the mechanisms by which viruses lose activity during droplet formation and drying is of great importance to understanding the spread of infectious diseases by virus-containing respiratory droplets and to developing thermally stable spray dried live or inactivated viral vaccines. In this study, we exposed suspensions of baculovirus, an enveloped virus, to isolated mechanical stresses similar to those experienced during respiratory droplet formation and spray drying: fluid shear forces, osmotic pressure forces, and surface tension forces at interfaces. DNA released from mechanically stressed virions was measured by SYBR Gold staining to quantify viral capsid disruption. Theoretical estimates of the force exerted by fluid shear, osmotic pressures and interfacial tension forces during respiratory droplet formation and spray drying suggest that osmotic and interfacial stresses have greater potential to mechanically destabilize viral capsids than forces associated with shear stresses. Experimental results confirmed that rapid changes in osmotic pressure, such as those associated with drying of virus-containing droplets, caused significant viral capsid disruption, whereas the effect of fluid shear forces was negligible. Surface tension forces were sufficient to provoke DNA release from virions adsorbed at air-water interfaces, but the extent of this disruption was limited by the time required for virions to diffuse to interfaces. These results demonstrate the effect of isolated mechanical stresses on virus particles during droplet formation and drying.

## Graphical Abstract

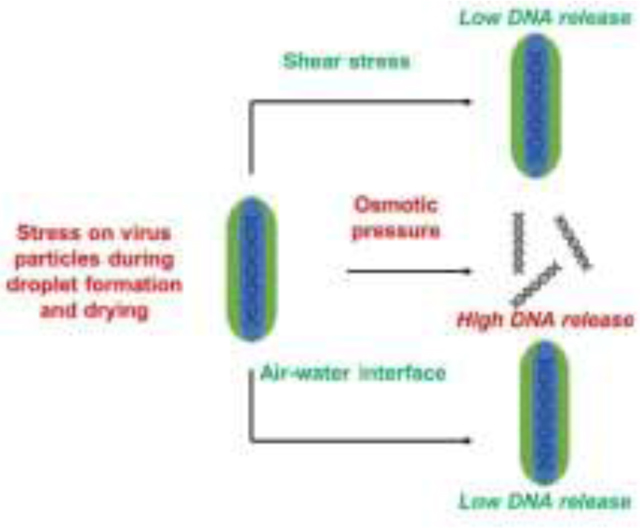

## Introduction

The COVID-19 pandemic focused attention on the importance of airborne transmission of viruses in aerosols.^1,2^ Formation and expulsion of microdroplets containing virus particles occurs when an infected person coughs, speaks, or sneezes, potentially resulting in airborne transmission of viruses or in fomite transmission of viruses from surfaces on which the droplets settle.^1^ In both cases, the rate at which aerosolized virions lose infectivity as the expelled microdroplets dry is of critical importance to their potential for transmission.

The stability of viruses during drying of aerosols is also of importance in spray drying, an industrial process widely used in the food and pharmaceutical industries wherein bulk solutions are aerosolized to form microdroplets that dry to form solid particles as the solvent evaporates when contacted by dry gas.^3,4^ Because spray drying has the potential to form products with enhanced thermostability, there is interest in developing spray dried formulations of live viral vaccines to alleviate vaccine cold-chain requirements, improve product shelf-life, and stabilize formulations of live viruses for cancer immunotherapies.^5–8^ Thus, understanding the mechanisms by which viruses may be destabilized during microdroplet formation and drying may provide insights into how viruses survive to spread disease, and also into the mechanisms of viral activity loss during spray drying of vaccines.

Mechanical stresses encountered during droplet formation and drying may impact virus stability. For example, fluid shear forces are exerted on suspensions of virions in saliva when actions like coughing and sneezing form and expel microdroplets.^9,10^ Likewise, during atomization processes used in spray drying, high shear, two-fluid nozzles typically are used to create sprays of fine droplets.^11^ The shear rates applied during droplet generation and expulsion caused by coughing or sneezing, or occurring during atomization in spray drying processes, are higher than those typically found in other biopharmaceutical manufacturing operations such as pumping, stirring, and tangential flow filtration which have been shown to have low impacts on viral infectivity loss.^12–14^ The shear rates associated with spray drying nozzles in particular have been suggested as a potential source of damage for virus particles during spray drying.^6,11^ In contrast, Morgan et al. tested spray dried formulations of adenovirus, a non-enveloped virus, and found that viral titer loss did not correlate with shear rates in the spray dryer nozzle.^6^ The effects of shear on viral stability have not been systematically evaluated for enveloped viruses.

The rate of evaporation of water from microdroplets and the concomitant increase in solute concentrations within the droplets depends on environmental conditions such as relative humidity and temperature, which can in turn affect the rate and extent of virus inactivation in droplets.^15,16^ Expelled saliva droplets are typically more than 90% water.^17^ They also contain a number of solutes in addition to virus particles, including mucus, salts, electrolytes, proteins, sugars, and cells.^17,18^ Formulations of viral vaccines developed for spray drying typically comprise sugars, buffer salts, and other excipients that contribute to particle formation and virus stability in the dried state.^4,8^ In both cases, solute concentrations increase as water evaporates^17^, increasing the osmotic pressure in the phase external to the viruses and exerting hyperosmotic pressure on virus particles. The resulting osmotic pressure differences between the solution exterior to the viral envelope and the capsid may exert sufficient force to disrupt viral envelopes or capsids and cause concomitant loss of infectivity.^19^ Additionally, in humid environments, expelled respiratory droplets may grow via the condensation of atmospheric water. The resulting reduction in solute concentrations in the liquid exterior to the lipid envelope^16^ would therefore result in hypoosmotic stress. Both hyperosmotic and hypoosmotic stress have been previously shown to damage viral particles, though the extent to which this happens varies with respect to the environmental or experimental conditions.^19–21^

Furthermore, it is possible that virus particles in droplets may diffuse to and adsorb at air-water interfaces (AWIs).^17^ Once adsorbed to such an interface, particles may be subjected to lateral surface tension forces that can be sufficient to rupture lipid membranes. Indeed, exposure to interfaces is known to be damaging to proteins and lipid vesicles.^14,22–24^

Herein we sought to estimate the magnitude of these mechanical forces to which an individual virion might be exposed during formation and subsequent drying of microdroplets. Theoretical, order-of magnitude estimates of mechanical forces were first determined, using droplet fluid properties, viral dimensions, and characteristic timescales corresponding to spray drying formulated baculovirus (BV) suspensions. We then subjected BV, as a model enveloped virus, to isolated shear, osmotic pressure, and interfacial forces of magnitudes characteristic of those exerted during droplet formation and drying. Following application of these forces, we monitored DNA release from viral capsids as a measure of capsid disruption. We found that osmotic pressure changes during drying had the potential to fully disrupt viral capsids, while fluid shear forces were less damaging. Interfacial forces were sufficient to cause DNA release from capsids, but the extent of damage was limited by the time required for virions to diffuse through bulk liquid within droplets and adsorb onto droplet air-water interfaces. These results represent the first systematic study of the effect of isolated mechanical stresses on enveloped virus particles, which leads to a fundamental understanding of how viruses are inactivated during processing.

## Experimental methods

### Viral stock.

Recombinant BV expressing green fluorescent protein was purchased from the University of Iowa Viral Vector Core. Sf9 cells were used to produce the BV working stock (2.3 × 10^8^ pfu/mL) by the University of Colorado Anschutz Medical Campus Cancer Center Cell Technologies Shared Resource Core Facility (RRID:SCR_021982). BV particles were suspended in Sf-900 III SFM from Thermo Fisher Scientific (Waltham, MA).

### Reagents.

Trehalose was purchased from Pfanstiehl (Waukegan, Illinois). All buffer salts were of reagent grade or higher. L-proline, l-glycine, tris(hydroxymethyl)aminomethane (tris), ethylenediaminetetraacetic acid (EDTA), SYBR Gold Nucleic Acid Gel Stain (10,000x concentrate), Qubit 1x dsDNA BR Lambda Standard, nuclease-free water, and polysorbate 80 (PS80) were all purchased from Thermo Fisher Scientific (Waltham, MA). USP 5% human serum albumin was donated to the lab by KBI Biopharma Inc. (Boulder, CO).

### DNA quantification by SYBR Gold staining.

Free DNA in solution was quantified using a SYBR Gold staining technique.^25^ Standard curves were made by diluting DNA standard in each buffer used in each experiment to account for strong dependence of SYBR Gold signal on individual buffer components.^25^ The ratio of sample DNA to thermally stressed sample DNA, after subtracting residual DNA in solution and correcting for dilution, was reported as ***% DNA release*.** Free DNA in unstressed BV samples was measured as a negative control to determine residual DNA levels derived from host cells used in virus production. BV suspensions that were thermally stressed in the presence of non-ionic surfactants^26^ were used as positive controls to determine DNA release upon total capsid disruption. For this, BV was incubated in 1x TE buffer (10 mM tris, 1 mM EDTA in nuclease free water, pH 7.5) with 2% PS80 for 3 hours at 60°C while shaking at 100 rpm [Fig. S1]. All samples were stained with 20 μL of 10x SYBR Gold in a black well-plate, with the total sample volume brought to 200 μL with 10% trehalose in 1x TE as necessary. Measurements were made approximately 5 minutes after the addition of SYBR Gold. DNA standards were run in duplicate and experimental samples and controls were run in triplicate.

### Osmolarity measurements and calculations.

Solution osmolarity (Π) was calculated as the sum of each component’s osmolarity $\left(\pi_{i}\right):\Pi =\sum \pi_{i}$. The contributions of l-proline or l-glycine at concentrations of 0.3, 1.0, 1.7, 2.0, and 2.5 M l-proline or l-glycine in 1x TE were calculated using $\pi_{i}=\phi_{i}n_{i}c_{i}$. Osmotic coefficients ($\phi_{i}$) for proline and glycine were determined from Smith and Smith and Tsurko et al., respectively.^27,28^ The contribution of 1x TE and the osmolarities of BV stock were measured by freezing point depression using an Advanced Instruments 3D3 Osmometer (Norwood, MA). The osmometer was calibrated with 100 and 1500 mOsM standards from Advanced Instruments. As an additional control, fresh USP 5% human serum albumin was measured between sample runs. Osmometry was performed in triplicate using a 200 μL sample. 0.3 M l-proline and l-glycine solutions in 1x TE were measured to confirm calculated values; however, l-proline and l-glycine solutions at a concentration of 1.0 M or higher could not be measured using this instrument. The change in osmolarity was calculated as the difference between calculated osmolarity of the solution compared to measured osmolarity of BV stock.

### Osmotic pressure effects on BV suspensions.

2.5 M l-proline and l-glycine solutions were prepared in 1x TE. The concentrated amino acid solutions were diluted with 1x TE in a 96-well plate to achieve a concentration of 0, 0.30, 1.0, 1.7, 2.0, or 2.5 M proline or glycine in 1x TE. In the assay well-plate, 20 μL BV was added to 20 μL SYBR Gold and 160 μL of the diluted amino acid solutions or 10% trehalose/TE (control). For this experiment, residual DNA was measured for BV diluted into 0.30 M l-proline or l-glycine in TE.

### Application of fluid shear stress to BV suspensions.

BV stock was diluted five-fold in 10% trehalose/TE. 2.5 mL of diluted viral stock was added to a Henke-Sass Wolf’s (HSW) Norm-Ject 30-mL syringe. Individual samples were pumped with a syringe pump at flow rates ranging from 0.20 to 20.0 mL/min through a Becton, Dickinson and Company (BD) 25G x 5/8” needle to achieve calculated average shear rates of 2.0 × 10^3^ to 2.0 × 10^5^ s^−1^ due to Poiseuille flow. Average shear stresses within the needle were calculated as $\frac{32\eta Q}{\pi D^{3}}$, where η is the dynamic viscosity, Q represents the flowrate, and D represents the inner diameter (0.26 mm) of the needle. Average shear rates were calculated by dividing the calculated average shear stress by 1.1 mPa s, the viscosity of a 10% trehalose solution^29^. These values yielded Reynolds numbers in the laminar flow regime (between 10 and 2000). As a control, one sample was loaded into the syringe but was not pumped through the needle. Approximately 2 mL of each pumped solution were collected in microcentrifuge tubes. All syringes and samples were kept on ice until use or assay. Samples were further diluted two-fold in 10% trehalose/TE before assaying. Percent DNA release was calculated compared to the positive control for total disruption. i.e., samples of BV incubated in TE/2% PS80 for 3 hr. at 60ºC.

### Ultrasonic atomization to create high surface area microdroplet mists.

BV stock or 10 ng/μL DNA standard (control) was diluted two-fold in 20% trehalose/2x TE buffer for a final solution of 10% trehalose/TE. 6 mL of diluted viral stock was added to a BD 50 mL syringe. Diluted viral stock was pumped at 1 mL/min through a SonoTEK 8700–120 MS, 120 kHz nozzle (Milton, NY) set at 2 W to produce droplets approximately 18 μm in diameter.^30^ Control samples were pumped through the nozzle without sonication enabled. Droplets were immediately collected in a 50-mL conical tube; the distance between the nozzle and the conical tube wall was adjusted so that the time between droplet formation and droplet coalescence as the droplets impinged on the tube walls was approximately 100 ms. Samples were kept on ice until use or assay. For this experiment, residual DNA was measured for diluted BV stock pumped through the nozzle at 0 W (no atomization), and samples of thermally-disrupted BV (suspensions of BV incubated in TE/2% PS80 for 3 hr. at 60C) were atomized to control for adsorptive losses of DNA on interior surfaces of the sonication system.

### Statistical analysis.

Significance was determined using the *t*-test function in Microsoft Excel^®^ to compare data before and after stress. Differences were considered significant at p-values less than 0.05.

## Results

### Estimated magnitudes of mechanical forces exerted on virions during microdroplet formation and drying.

**Figure 1 –:**
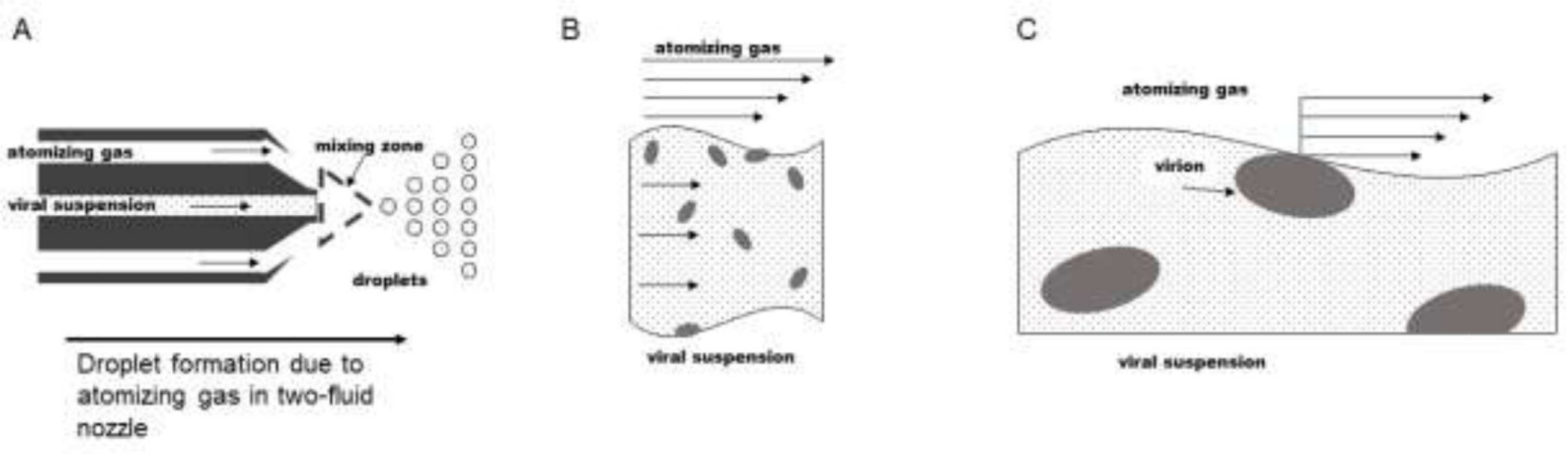
Shear stress due to the atomizing gas stream during atomization in a two-fluid nozzle with external mixing may damage virus particles. Nozzle, droplets, virions, and other solute molecules are not drawn to scale with respect to each other for visual purposes. A. A schematic of a typical two-fluid nozzle with external mixing. The atomizing gas stream typically flows at a significantly higher rate than the liquid feed. In the mixing zone, the gas stream accelerates the liquid stream, and high frictional forces from the gas stream disintegrate the liquid stream into droplets. B. Flow profiles for the atomizing gas and liquid feed which contains a suspension of viral particles (ellipsoids) and other excipients (small black dots). C. A virion near the boundary between the liquid feed and the atomizing gas can be subjected to shear stress.

**Figure 2 –:**
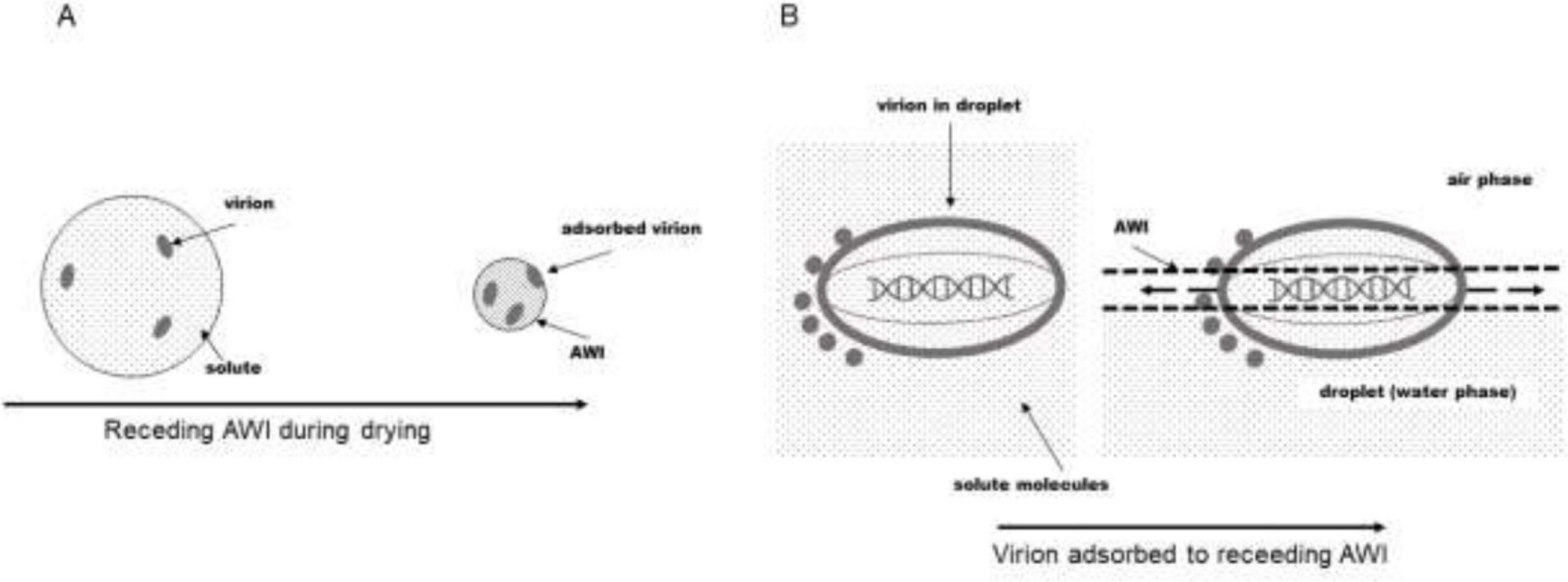
Viruses adsorbed to the AWI experience lateral forces due to surface tension. Droplets, virions, solute molecules, and the AWI are not drawn to scale for visual clarity. A. During drying, the droplet surface recedes as water is removed by evaporation. B. Virus particles may become adsorbed to the AWI. An equatorial band where the virus finds itself adsorbed will experience lateral forces (shown as dashed arrows within the AWI) due to surface tension at the interface.

**Figure 3 –:**
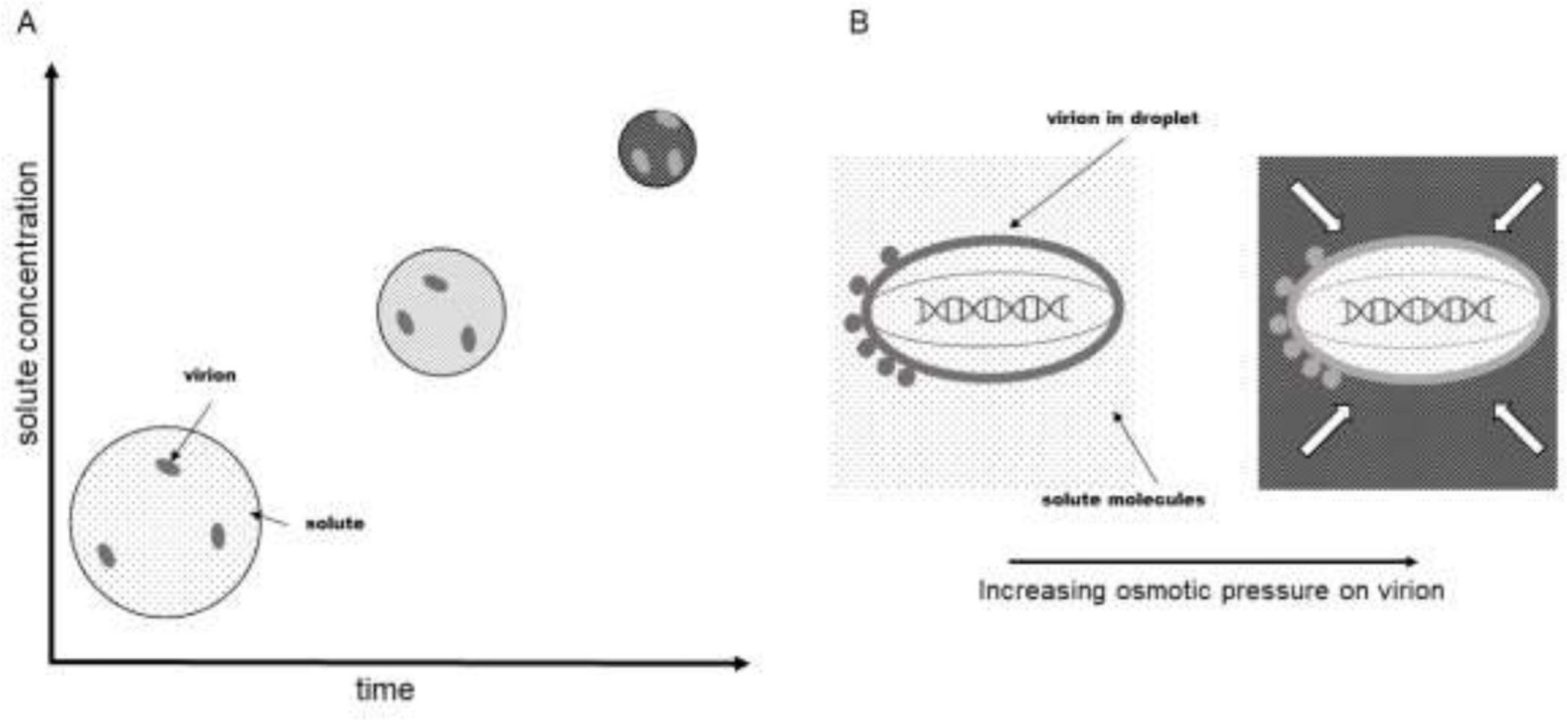
Increases in solute concentration as water is removed from droplets during drying exert increased osmotic pressures on virions. Droplets, virions, and solute molecules are not drawn to scale with respect to each other for visual purposes. A. During the course of droplet drying, water is removed by evaporation and the solute concentration increases. B. Viral capsids are generally permeable to water and salts, but not to other molecules that might be present.^17,19^ Thus, during drying, the external solute concentration increases compared to the solute concentration within the virus particle, inducing hyperosmotic stress on the particle isotopically, shown by the white arrows.

Viruses can be subjected to multiple mechanical stresses during droplet formation and drying [Fig. 1–3]. For example, droplets experience transient shear stresses during droplet formation from bulk fluid. As reported by Morgan et al.,^6^ atomization in two-fluid nozzles [Fig. 1] is associated with shear rates ranging from 10^2^ to 10^5^ s^-1^. During drying, viruses adsorbed to the air-water interface (AWI) of the droplet during drying may experience surface tension stresses [Fig. 2]. Additionally, the change in solute concentration can induce osmotic pressure stresses on viruses in the droplet [Fig. 3]. To form hypotheses about which of these mechanical stresses might be the most detrimental to viral capsid integrity and titer, we performed order of magnitude estimates for the characteristic pressures or forces that viral particles would experience during droplet formation and drying. For these calculations, we used the physical dimensions of our model enveloped virus BV (approximately 100 nm wide and 200 – 450 nm long)^31^, giving a surface area on the order of 10^−13^ m^2^ for the roughly ellipsoidal virion).

Shear rates typically experienced during biopharmaceutical industrial operations such as pumping and stirring (10^1^ to 10^3^ s^−1^) do not negatively impact viral titers in pharmaceutical formulations.^12,13,23^ However, because droplet formation during spray drying requires higher shear rates (10^2^ to 10^5^ s^−1^), it has been suggested that shear might be a source of damage to viral particles during spray drying.^12,13^ Since shear rates experienced during spray drying are larger than those experienced in coughing or otherwise expelling droplets, we considered the maximum shear rate a virus might experience during droplet formation in spray drying as an upper limit.^9^ To do this, we used parameters for drying conditions characteristic of a pilot scale spray dryer using a two-fluid nozzle with external mixing.^6^ As shown in Fig. 1A, this nozzle design passes a concentric stream of an atomizing gas around the fluid stream. The liquid and gas streams meet in the mixing zone just beyond the nozzle outlet, shown with the dashed triangle in Fig. 1A. Typically the gas stream flows at a significantly higher linear velocity than the liquid stream [Fig. 1B].^11^ As the gas stream meets the liquid jet, it accelerates the liquid stream to the average or mixing rate of the streams.^11^ The frictional forces acting on the liquid surface are high and cause the liquid to break up into droplets [Fig. 1B].^11^ Virus particles near the boundary between the liquid and gas streams during the atomization process experience the highest shear stresses [Fig. 1C].

In order to determine an upper boundary for the amount of shear that might be experienced during the atomizing process, we estimated a characteristic shear rate (γ) generated during atomization in a two-fluid atomizing nozzle based on an atomizing gas velocity (v) of approximately 260 m/s and a nozzle inner diameter ($D_{i}$) of 0.7 mm using $\gamma =\frac{2\left(v_{mixing}-v_{liquid}\right)}{D_{i}}$.^6^ When (as in the present case) the liquid flowrate is slow compared to the gas flowrate, $v_{mixing}-v_{liquid}\approx v_{gas}$. ^6^ This gives characteristic shear rates on the order of 10^5^ s^-1^. Shear stress experienced by a virus particle at the interface between the liquid stream and atomizing gas was calculated by multiplying this shear rate by viscosity of the atomizing nitrogen gas, 1.8 × 10^−5^ Pa s. Shear force exerted on a virus particle by this atomizing gas was estimated by multiplying shear stress by virus surface area. This gave an expected force on the order of 1 pN [Table 1]. The timescale for atomization, 10^−5^ s, was estimated by calculating the time for an individual droplet to form during spray drying by dividing the time of a spray dry run by the number of particles formed during the spray dry run. A virion adsorbed to the AWI of a droplet might experience lateral tensile forces due to surface tension [Fig. 2].

An estimate of the lateral tensile force exerted on interfacially adsorbed virions by surface tension [Table 1] was calculated as F=σ2πr using the surface tension (σ) of water (70 mN/m) and the circumference of an adsorbed virion with a radius (r) of 200 nm resulting in a force on the order of 10 nN.^32^ Notably, this estimated tensile force is larger than forces necessary to unfold proteins (20 – 150 pN)^23,33^ and rupture lipid vesicles (30 – 70 pN)^34^, and roughly on the order of compressive forces (a few nN) reported by Ivanovska et al. to cause rupture of bacteriophage capsids in nanoindentation experiments with atomic force microscopy.^35^ Thus, surface tension forces exerted on virions at the AWI might be enough to damage some or all of their structural components. Because the lateral tensile force on a virion embedded within the AWI acts on a circumferential band on the virion surface with an area 2πrh [Fig 2B], we estimated the pressure exerted on this circumferential band as σ/h using the air-water surface tension and an interface thickness h of approximately 2 nm^32^ to be approximately 10 MPa. These lateral forces due to surface tension would only occur in the presence of an AWI, and thus would only be of importance while the droplet was drying over the course of the characteristic time required for droplet evaporation of ~100 ms.^4^

The change in osmotic pressure in the medium surrounding a virus particle in a drying droplet was calculated for a solution as solute concentrations were increased from 10% trehalose (290 mOsM) to approximately 75% trehalose (2300 mOsM) since the latter concentration is approximately the trehalose concentration at which onset of glass formation occurs during spray drying.^36^ Pressure change (ΔP) was calculated from the van’t Hoff relationship by ΔP=RTΔc using T = 298K, yielding ΔPvalues on the order of 1 MPa [Table 1]. Pressure was multiplied by virus particle surface area to determine the force exerted on the lipid membrane, which was on the order of 10^−7^ N. Notably, this is greater than the force that is reported to damage viral capsids by nanoindentation.^35^ Again, this force would only be experienced during the characteristic ~100 ms time required for drying.^4^

From this analysis [Table 1], it is clear that both surface tension and osmotic pressure forces and stresses experienced by a virion are substantially larger in magnitude than the shear stress due to atomization. Moreover, surface tension and osmotic pressure forces are applied for much longer periods of time than is shear stress. Therefore, surface tension and osmotic pressure are expected to be more damaging than shear stress overall. It is not immediately clear, however, whether one would expect surface tension or osmotic pressure to be the dominant source of damage. Roughly speaking, the stress/pressure and forces applied are relatively similar in magnitude. Moreover, the forces are applied in very different ways. For example, interfacial tension results in a tensile stress that is applied along a lateral band on the virion at the air-water interface [Fig. 2B], whereas osmotic pressure differences cause an isotropic radial stress [Fig. 3B]. The ability of a virion to withstand these two types of stress may be very different. Therefore, we subjected virus particles to these stresses in isolation to test these predictions experimentally.

### Osmotic pressure effects of DNA release from BV suspensions.

Measured osmolarity values are listed in Table 2 with the predicted values for 0.30 M l-proline or l-glycine in 1x TE. Calculated and measured osmolarities for 0.30 M l-glycine and l-proline in 1x TE were similar.

**Figure 4 –:**
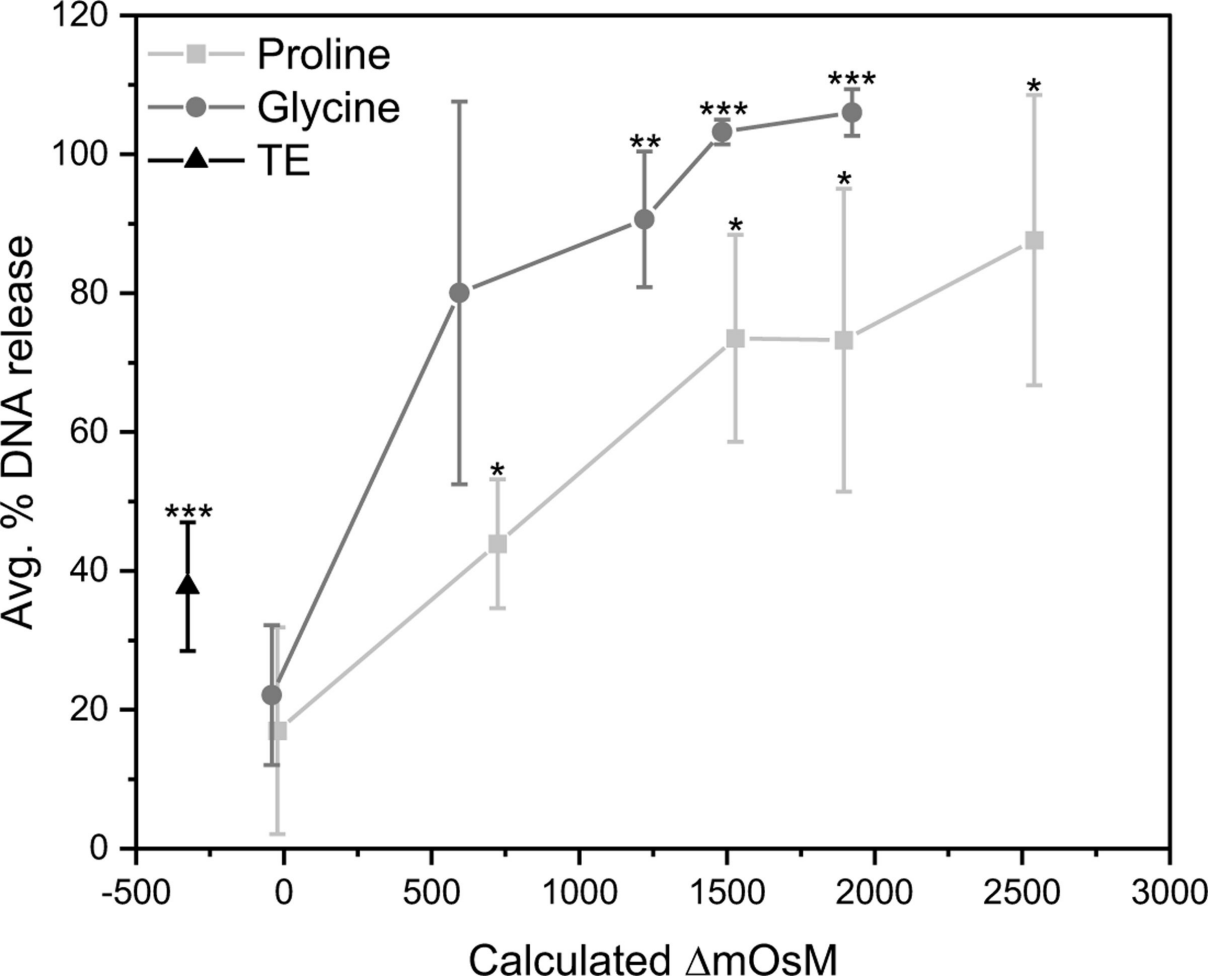
Percent DNA release versus change in solution osmolarity. Note that 0.30 M solutions of glycine and proline in 1x TE were not exactly iso-osmotic with the control (10% trehalose in 1x TE), possibly contributing to the moderate, though statistically insignificant, DNA loss seen near ΔmOsM of zero. Asterisks denote significance for sample compared to DNA release into 10% trehalose solution. * p-value < 0.05, ** p-value < 0.01, *** p-value < 0.001. Error bars represent the standard deviation of three independent replicates.

BV stock solutions were diluted into hypoosmotic (1x TE with no amino acid) or increasingly hyperosmotic solutions (up to 2.5 M l-proline or l-glycine in 1x TE). BV stock was also diluted into an approximately iso-osmotic solution of 10% trehalose in TE as a control. Dilution into a hypoosmotic solution resulted in approximately 40% DNA release [Fig. 4]. In l-proline and l-glycine solutions, DNA release increased with increasing osmotic pressure difference, up to near total DNA release in the high osmolarity solutions [Fig. 4]. Notably, hyperosmotic stress was found to have the greatest impact on capsid disruption compared to shear stress and atomization, as described further below.

### Effects of Shear stress on viral stability.

**Figure 5 –:**
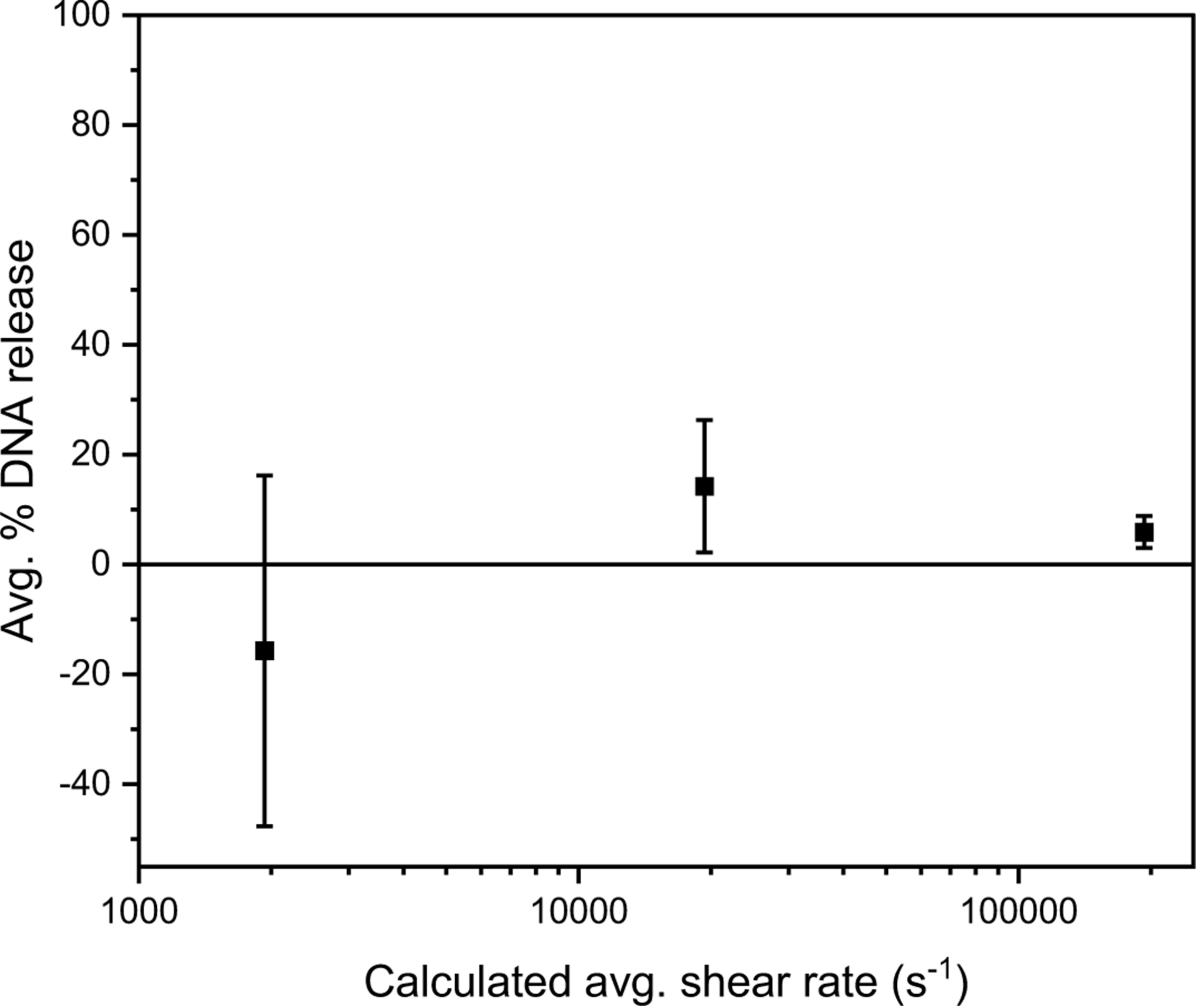
Percent DNA release as a function of shear rate. All values were not significantly different from the unstressed control (p-value > 0.1). Error bars represent the standard deviation of three independent replicates. The apparent negative value for DNA release was due to background subtraction.

Diluted BV stock was pumped through a capillary at 0.20, 2.0, or 20.0 mL/min to achieve average shear rates of 2.0 × 10^3^, 2.0 × 10^4^, or 2.0 × 10^5^ s^−1^, which are in the range of expected characteristic shear rates of two-fluid nozzles in spray drying.^6^ Less than 15% DNA release was seen for all samples, with no clear trend for percent DNA release with respect to the calculated shear stress [Fig. 5]. Additionally, DNA release was not statistically significant (p-value > 0.1) at each shear rate compared to an unstressed control. Although high shear rates have been suggested to be a source of significant titer loss in spray drying operations, little effect of high shear stress on BV viral capsid disruption was observed in this experiment [Fig. 5].

### Viral damage at droplet air-water interfaces.

The goal of this experiment was to isolate the effect of AWI exposure on particles. To do this, mists of microdroplets containing BV were created using an ultrasonic nozzle and were suspended in air for approximately 100 ms before they impinged and coalesced on the walls of a conical tube, a time chosen to mimic the typical lifetime of a droplet under spray drying conditions.^4^ This created droplets with a high specific surface area to which virions could diffuse and adsorb, but the droplets were allowed to coalesce and were collected before significant drying (and associated changes in osmotic pressure) could occur. Diluted BV stock was atomized at 2 W using a SonoTEK 8700–120 MS, 120 Hz nozzle, generating microdroplets of approximately 18 μm in diameter^30^, similar in size to those formed during spray drying. Samples of the coalesced droplets were collected from the conical tube and stained with SYBR Gold in a well-plate for DNA release measurement. 21% ± 10% DNA release was measured for the atomized viral samples (not significantly different from the unstressed control, p-value > 0.1). This suggested that some viral particles may have been disrupted during atomization due to exposure to the AWI, but most viral particles in solution were not damaged.

## Discussion

Based on the orders of magnitude of forces encountered during droplet formation and drying, we predicted that osmotic pressure forces and interfacial forces might be more damaging during drying than the shear forces involved in droplet formation. Indeed, we found that under experimental conditions designed to mimic those occurring during typical spray drying operations, osmotic pressure forces were more disruptive to viral capsids than those arising from hydrodynamic shear or from surface tension effects. Virion damage following exposure to the AWI, or to fluid shear forces applied at levels typical in two-fluid spray drying nozzles was statistically undetectable, in contrast with damage due to exposure to osmotic pressure changes associated with microdroplet drying.

We had expected, based on our theoretical order-of-magnitude force estimates, that surface tension forces at droplet AWIs would provide a more potent source of damage during droplet formation and drying than forces arising from fluid shear stresses. However, interfacial exposure in the microdroplet mists created by atomization resulted in at most only a small increase in the fraction of BV virions releasing DNA. This may have been due to the relatively small fraction of the virions within a microdroplet that were able to diffuse to and adsorb at the droplet AWI during the time between droplet formation and collection. The diffusion coefficient (D) for BV in a 10% trehalose solution with a viscosity (η) of 2.5 × 10^−4^ Pa s^37^ can be estimated as approximately 9 μm^2^/s using the Stokes-Einstein relationship $D=\frac{k_{b}T}{6\pi \eta r}$. Droplets traveled for approximately 100 ms before coalescing at the wall of the conical collection tube, and a characteristic diffusion length of 2 μm was calculated for this time by $L=\sqrt{4Dt}$. A spherical shell with a thickness of 2 μm at the outer edge of a droplet would comprise approximately 50% of the droplet total volume, suggesting that if virus particles were well mixed in the bulk solution before droplet formation, only about half of the virus particles at maximum could migrate to the air water interface within 100 ms, the approximate time required for droplet evaporation during spray drying.^4^ This does not account for the fact that the increase in concentration of trehalose or other excipients during drying will increase the viscosity, further decreasing the diffusion rate throughout the droplet. Once virions diffuse to the interface, incorporation of virions to the interface requires additional time.^38^ Additionally, incorporation of virions into the AWI may be further inhibited by surface active components in the media^17^ or slowed due to electrostatic barriers experienced by the virions at the AWI,^39^ and the extent to which the virion is attracted to the AWI depends on components and pH of the droplet as well as properties of the virus such as its hydrophobicity and surface charge.^17^ Overall, even though forces at the AWI (both due to surface tension or electrostatic effects) may be sufficient to damage virions, the diffusive, kinetic, and electrostatic barriers to virion adsorption at the AWI suggest that droplets dry before there is sufficient time for significant adsorption and associated virion damage to occur.

Virus half-life has been shown to have a U-shaped dependence on relative humidity at a given temperature.^16,40,41^ In extremely humid environments where little evaporation occurs, and in dry environments where droplets dry quickly, virus viability is enhanced compared to intermediate humidities.^16,41^ Viruses have been shown to be infectious long after droplets have dried on surfaces.^41^ In our experiments, we tested for viral DNA release after applying osmotic pressure differences across BV envelopes by diluting into hyper- or hypoosmotic solutions and allowing the BV to be exposed to these conditions for 5 minutes. This exposure time is less than what viruses might experience during spray drying operations, where drying to particles with moisture contents near 1% requires only approximately 100 ms.^4^ This exposure time is an intermediate value for what viruses might experience in suspended respiratory droplets, which might remain in air for a few seconds or over 30 minutes depending on ambient temperature and humidity, as well as whether the surrounding air is moving or relatively still.^16,42^

In AWI exposure experiments, we were able to match the timescale of droplet formation and AWI exposure to what occurs during spray drying. However, in expelled respiratory droplets, it is possible that they may remain suspended for longer periods of time based on their size and the ambient humidity,^17,43^ allowing more virus particles to move and adsorb to the surface. It is possible extended time for AWI exposure may lead to greater damage to viral capsids.

An additional rationale for investigating the impact of stress on viral capsid disruption is to provide insight into the type of damage that might be implicated in viral titer loss. Viral capsid integrity and DNA retention is necessary for viral infectivity and production of infectious viral particles in the cell. SYBR Gold staining allowed us to quantify the DNA released or exposed in solution to specifically assess one type of damage occurring during these processes. However, other types of damage (not resulting in DNA release) could also deactivate virus particles. For example, it is possible that mechanical stresses might damage other viral structural components including surface proteins and viral envelopes, which might result in loss of viral infectivity without capsid disruption and DNA release. Notably, the viral envelope has been shown to be less stable than viral capsids on surfaces and during desiccation.^44^ A further investigation of the effect of these stresses on other viral components using orthogonal characterization methods would give a more complete picture of sources of loss of viral activity during droplet formation and drying.

## Conclusion

Here we present the first systematic study of the effect of isolated mechanical stresses on enveloped virus particles to better understand how viruses are inactivated during droplet formation and drying. Overall, theoretical analyses suggest that, during drying of virus-containing microdroplets, increases in solute concentrations in liquids external to the viral envelopes may cause osmotic pressure differences, exerting forces on the virions that are similar to those that might be experienced due to surface tension at the interface. Both osmotic forces and surface tension forces are substantially larger than those expected due to fluid shear forces. Surface tension forces appear somewhat less likely than osmotic pressure changes to impact viral capsid integrity, due to a number of barriers that can prevent virions from adsorbing to the AWI. These analyses were confirmed experimentally using DNA release from baculovirus as a metric for capsid disruption. Neither fluid shear during droplet formation in high-shear, two-fluid nozzles nor exposure of baculovirus to surface tension forces at droplet air-water interfaces caused significant DNA release, whereas damage to BV virions correlated directly to osmotic pressure changes.

Our studies, combined with empirical observations of increased retention of viral infectivity in droplets in either very low or very high humidity environments, suggest that a strategy for preserving maximum viral activity in dried preparations created for industrial applications would be to dry liquid formulations as quickly as possible so as to limit the exposure time of viral particles to rapidly changing osmotic concentrations. Spray drying, using small initial droplet sizes and drying gases with relatively high temperatures and low humidity may be one way to achieve the very fast drying times needed to limit osmotic pressure induced damage to viral particles. Very slow drying, which might allow for equilibration of osmotic pressure across envelop membranes, might also preserve viral activity as microdroplets dry, but likely would result in unacceptably low throughput in an industrial process. Conversely, if the goal is to reduce viral viability, controlling environmental humidity within ranges where droplets dry quickly enough to cause rapid changes in trans-membrane osmotic pressure, but slowly enough to expose the viral particles to damaging osmotic pressure gradients for sufficiently long times may limit virus’ survival in expelled aerosols.

## Supplementary Material

1

## Figures and Tables

**Table 1-:** Estimated mechanical stresses experienced by baculovirus particles during drying.

Source of stress	Pressure or stress (Pa)	Force (N)	Exposure time (s)
Shear due to atomization	10^1^	10^−12^	10^−5^
Surface tension at droplet interface	10^7^	10^−8^	10^−1^
Osmotic pressure change during drying	10^6^	10^−7^	10^−1^

**Table 2-:** Measured osmolarity of listed solutions.

Solution	Measured osmolarity (mOsM)	Calculated osmolarity (mOsM)
BV stock	350.3 ± 3.4	n/a
1x TE	24.0 ± 0.0	n/a
1x TE, 10.0% trehalose	294.0 ± 1.4	n/a
1x TE, 0.30 M glycine	323.3 ± 4.7	310
1x TE, 0.30 mM proline	348.5 ± 2.5	330

## Highlights

- Mechanisms for virus inactivation in drying explored
- Drying-induced osmotic pressure changes disrupted viral capsids
- Damage to viral capsids by surface tension forces limited by kinetics of interfacial adsorption
- Fluid shear forces relatively unimportant to viral inactivation during droplet drying
