# Supplementary material for: Effect of mechanical stresses on viral capsid disruption during droplet formation and drying
Source: Colloids Surf B Biointerfaces. Author manuscript; Available in PMC 2024 Apr 2. (PMC10986848; doi:10.1016/j.colsurfb.2023.113661)
Supplement: 1 [file NIHMS1950275-supplement-1.pdf]

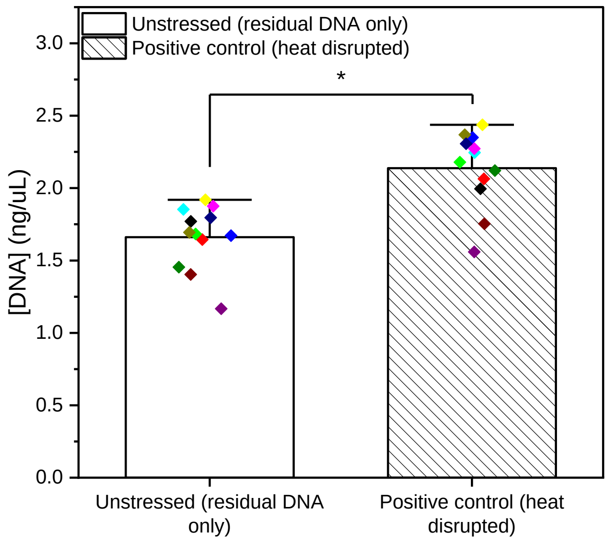

Figure S1 - Free residual DNA in unstressed viral stock vs. total DNA in heat disrupted sample. Increase in DNA is statistically significant (p-value < 1E-06). Colored symbols denote measurements from the same experiment. Data points are offset for visual clarity. The top line of each box represents the mean value of the dataset, and the error bar represents the data range within 1.5 times the interquartile range.
